# Protocole of a controlled before-after evaluation of a national health information technology-based program to improve healthcare coordination and access to information

**DOI:** 10.1186/s12913-017-2199-y

**Published:** 2017-04-21

**Authors:** Florence Saillour-Glénisson, Sylvie Duhamel, Emmanuelle Fourneyron, Laetitia Huiart, Jean Philippe Joseph, Emmanuel Langlois, Stephane Pincemail, Viviane Ramel, Thomas Renaud, Tamara Roberts, Matthieu Sibé, Frantz Thiessard, Jerome Wittwer, Louis Rachid Salmi, for the EvaTSN Research group

**Affiliations:** 1 ISPED, Centre INSERM U1219 Bordeaux Population Health Research Center, University Bordeaux, 146 rue Léo Saignat, 33076, F-33000 Bordeaux, France; 2 INSERM, ISPED, Centre INSERM U1219 Bordeaux Population Health Research Center, 146 rue Léo Saignat, 33076, F-33000 Bordeaux, France; 3 CHU de Bordeaux, Pôle de Santé Publique, Service d’Information Médicale, 146 rue Léo Saignat, 33076, F-33000 Bordeaux, France; 4 Département de Médecine Générale, Université de Bordeaux, 146, rue Léo Saignat 33076, Bordeaux, Cedex France; 5 Institut de Santé Publique d’Epidémiologie et de Développement, Université de Bordeaux, 146, rue Léo Saignat 33076, Cedex F-33000 Bordeaux, France; 6 CHU, Unité de Soutien Méthodologique, Saint-Denis, F-97400 France; 7 University Bordeaux/Sciences Po Bordeaux, Centre Emile Durkheim, F-33000 Bordeaux, France; 8 Groupe Hospitalier Est Réunion, Route Nationale 3 30, 97470, Saint-Benoît-de-Carmaux, France; 9 Centre Emile Durkheim, Science Politique et Sociologie Comparatives, Université de Bordeaux, 3Ter, Place de la Victoire 33076, Bordeaux, Cedex France

**Keywords:** Health information technology, Program evaluation, Patient care management

## Abstract

**Background:**

Improvement of coordination of all health and social care actors in the patient pathways is an important issue in many countries. Health Information (HI) technology has been considered as a potentially effective answer to this issue. The French Health Ministry first funded the development of five TSN (“Territoire de Soins Numérique”/Digital health territories) projects, aiming at improving healthcare coordination and access to information for healthcare providers, patients and the population, and at improving healthcare professionals work organization. The French Health Ministry then launched a call for grant to fund one research project consisting in evaluating the TSN projects implementation and impact and in developing a model for HI technology evaluation.

**Methods:**

EvaTSN is mainly based on a controlled before-after study design. Data collection covers three periods: before TSN program implementation, during early TSN program implementation and at late TSN program implementation, in the five TSN projects’ territories and in five comparison territories. Three populations will be considered: “TSN-targeted people” (healthcare system users and people having characteristics targeted by the TSN projects), “TSN patient users” (people included in TSN experimentations or using particular services) and “TSN professional users” (healthcare professionals involved in TSN projects). Several samples will be made in each population depending on the objective, axis and stage of the study.

Four types of data sources are considered: 1) extractions from the French National Heath Insurance Database (SNIIRAM) and the French Autonomy Personalized Allowance database, 2) Ad hoc surveys collecting information on knowledge of TSN projects, TSN program use, ease of use, satisfaction and understanding, TSN pathway experience and appropriateness of hospital admissions, 3) qualitative analyses using semi-directive interviews and focus groups and document analyses and 4) extractions of TSN implementation indicators from TSN program database.

**Discussion:**

EvaTSN is a challenging French national project for the production of evidenced-based information on HI technologies impact and on the context and conditions of their effectiveness and efficiency. We will be able to support health care management in order to implement HI technologies. We will also be able to produce an evaluation toolkit for HI technology evaluation.

**Trial registration:**

ClinicalTrials.gov ID: NCT02837406, 08/18/2016.

## Background

### The challenges of Health Information Technologies to improve healthcare coordination

All the health systems face challenges in delivering high-quality, effective and safe care at an affordable cost. Improvement of coordination of all health and social care actors in the patient pathways is an important issue in many countries [1–3]. Healthcare coordination is defined by the Agency for Healthcare Research and Quality as “the deliberate organization of patient care activities between two or more participants (including the patient) involved in a patient’s care to facilitate the appropriate delivery of health care services” [4]. Care coordination is thus the patient-centred organization of care that providers (including patient caregivers) should share to improve the quality of a patient’s management and, ultimately, the patient’s health [5–8]. The development of ambulatory care and the scattering of healthcare producers lead to increasing risk of care fragmentation [9]. Moreover, in the context of the increase in life expectancy and of the number of patients with chronic illnesses, continuity of care becomes a crucial condition for care quality and security [10, 11]. A key success factor to care coordination is sharing of the same holistic view of a patient’s condition by all actors of hospital, ambulatory and medico-social sectors, including the patient’s active diseases and current treatments, and the planned care pathway that establishes the role of each provider. Unfortunately, transitions from one setting or provider to another still frequently lack clear communication and coordination, resulting in an increase in healthcare costs and reduced quality of care including polypharmacy and adverse drug interactions, duplication of services, unnecessary emergency department utilization, high hospital readmissions rates, and in the worst cases patient injury [5, 9, 12, 13].

Given the large volume of transactions in the system and the need for new evidence-based practice and other information management activities, Health Information (HI) technology has been considered as a potentially effective answer to this issue [12, 14]. A recent WHO (World Health Organization) report shows that there is around Europe tangible progress in the mainstreaming of technology solutions to improve public health and health service delivery [15]. At the core of this technology-led transition is an adjustment in the way health information is captured, viewed, processed, exchanged and stored. HI technology involves a broad group of activities that use electronic means to deliver social and health-related information, resources and services.

HI technologies are potentially effective tools to improve information system between healthcare and social care professionals and between patients and health care professionals, allowing a better access to more precise information, improving communication and coordination between healthcare and social care organizations and healthcare professionals. They may allow the centralisation of all patient management information, the creation of shared tools for healthcare coordination, the creation of alerts, feedback, reminders thus favouring the implication of health and social care professionals and patients in their care pathway, continuous quality assessment and care efficiency as well as the development of a shared culture between professionals [15].

Literature on HI technology efficacy and efficiency shows controversial results. Even if many studies show a real but modest effect [16–20], others suggest the absence of positive effect, particularly on outcomes and cost-effectiveness indicators [17, 21–23], acknowledging a gap between the postulated and empirically demonstrated benefits of HI technologies [21, 24]. Some studies even indicate potential risks and unintended effects [25, 26].

These controversial results are explained by methodological reasons (global poor quality of the studies, heterogeneity of evaluated HI technologies and of HI technologies implementation's context, hampering the external validity of the studies) but also by pitfalls in HI technology development, often disregarding the interdependencies between technology, human characteristics and the socioeconomic environment [27–31]. Many researchers advocate larger, well-designed, controlled studies evaluating HI technology against a comprehensive set of measures, ideally throughout all stages of the technology’s life cycle [32, 33]. Such evaluation should be characterised by careful attention to socio-technical factors and organizational issues to maximise the likelihood of successful implementation and adoption [25, 26]. Another key issue for the development of HI technology is the political commitment, backed by sustainable funding. Published evidence of the information needed to make decisions about acquiring and implementing HI technology in different settings is nearly inexistent [15].

### Healthcare coordination and HI technology development in France

In France, rules have been developed since the late 1990s to introduce healthcare system change for healthcare coordination improvement [34]. General Practitioners (GPs) have gained a major role in care coordination, thanks to a semi gate-keeping system that provides incentives to people to visit their GP prior to consulting a specialist. A coordinated care pathway was implemented with higher co-insurance for patients consuming care out of this pathway and new categories of co-payment for patients were created with the introduction of deductibles on some categories of care such as drugs, physicians and nurses consultations or patient transportation. Creation of primary healthcare centres that bring together professionals from different specialties is encouraged to improve coordination of care and cooperation between healthcare professionals. However, major problems include a lack of coordination between hospital and ambulatory services, between private and public provision of care and between healthcare and public health [34].

In this context, French public authorities are supporting HI technologies development. In 2014, a governmental grant, called TSN (“Territoire de Soins Numérique”/Digital health territories), selected five HI technology development projects across five pilot areas containing between 200,000 and 500,000 inhabitants each in five French regions: Aquitaine, Bourgogne, Ile-de-France, la Réunion and Rhônes-Alpes. These HI technology projects are supposed to introduce changes in healthcare organization, aiming at improving healthcare coordination and access to information for healthcare providers, patients or the population and at improving healthcare professionals’ work organization.

In September 2014, the French Health Ministry launched a call for grant to fund one project that should develop a method to evaluate the five TSN projects’ use and implementation and their impact on three main outcomes: healthcare quality, healthcare professionals’ work organization and efficiency. The selected project should also be able to create a general framework for the evaluation of HI technology services.

### The EvaTSN project

This article presents the protocol of the project that was selected, called EvaTSN (Evaluation Territoire de Soins Numérique). It has been conceived by a multidisciplinary team composed of health services researchers, economists, sociologists, management researchers, HI technology researchers, epidemiologists and healthcare professionals. An operational team is in charge of the project lead and management.

The project’s main objectives consist in evaluating the TSN program implementation and impact and developing a model for HI technology evaluation. The specific objectives are structured around four axes: 1) TSN program implementation and use; 2) Impact of TSN program on patient pathway quality and safety, including access to care; 3) Impact of TSN program on healthcare professionals work organization; and 4) Economic sustainability and efficiency of the TSN program.

## Methods

### Design of the study

**Fig. 1:**
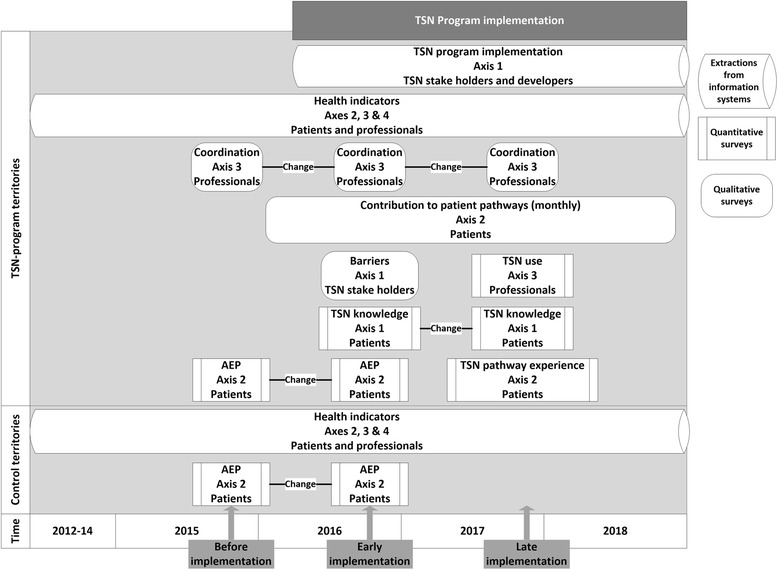
EvaTSN project data collection schedule and organization, France

The study is mainly based on a controlled before-after study design (Fig. 1). Data collection covers periods before TSN program implementation (2012-2015 or T0 period), during early TSN program implementation (2016, T1 period) and at late TSN program implementation (starting in 2017, T2 period), in the five TSN projects territories and in five comparison territories. Analyses of TSN program implementation and use are based on a before-after design.

### The interventions – the TSN projects

All the TSN projects develop digital services for healthcare users and healthcare and social care producers (hospitals, ambulatory sector, medico-social sector) to improve healthcare coordination, collaboration between professionals and access to healthcare and prevention information and professional guidelines (Table 1).

**Table 1:** Presentation of digital/E-services and functionalities in each TSN project area – EvaTSN project, France

Type of E Health Services	E Health-services functionalities	AQT	BGN	IF	LR	RHA
Organisation of healthcare coordination^a^	- Platform for service coordination	✓	✓	✓	✓	✓
	- Digital coordination service	✓	✓	✓	✓	✓
	- Call center for informal caregivers					✓
E-services for users/patients	- Diffusion service of general healthcare information	✓	✓	✓	✓	✓
	- Medical information management services	✓	✓	✓	✓	✓
	- Administrative information management services	✓	✓	✓	✓	✓
	- Community of connected patients				✓	
E-services for professionals	- General health information service	✓	✓	✓	✓	✓
	- Professionals' patient medical information management	✓	✓	✓	✓	✓
	- Administrative information management services	✓	✓	✓	✓	✓
	- Community of connected professionals		✓	✓		✓
Administrative and monitoring services for policy makers	- Administrative monitoring	✓	✓	✓	✓	✓
	- Epidemiological monitoring		✓		✓	✓
	- eHealth innovation promotion	✓	✓	✓	✓	✓
	- Interoperability management	✓	✓	✓	✓	✓

^a^between healthcare producers and between health care producers and patients, *AQT* aquitaine, *BGN* Bourgogne, *IF* Ile-deFrance, *LR* La Réunion, *RHA* Rhône-Alpes

Most TSN projects target patients with chronic conditions or elderly dependent people. Some of them focus on patients suffering from specific diseases, including congestive heart failure (Aquitaine, Bourgogne, la Réunion regions), cancer (Bourgogne, Ile-de-France regions), stroke (Bourgogne), respiratory failure (Aquitaine), or specific populations, including young people (Bourgogne), pregnant women (Ile-de-France), obese and disabled people (la Réunion), and situations of social vulnerability (Bourgogne, Ile de France).

### Study populations

Three populations will be considered: 1) “TSN targeted people”: healthcare system users and people having characteristics targeted by the TSN projects, 2) “TSN patient users” that is to say people included in TSN experimentations or using particular services and 3) “TSN professional users”, i.e. healthcare professionals involved in TSN projects. Several samples will be constituted in each population depending on the objective, axis and stage of the study.

### Outcomes and measurements – data sources and data collection

Four types of data sources are considered (Table 2).

**Table 2:** EvaTSN project data collection: indicators collected, data collection tools and study populations, France

Assessment/Indicators	Study populations	Type of data collection	Data collection tool
Axis 1 – TSN projects implementation			
Knowledge of TSN projects	HS^d^ users	Ad hoc survey	Ad hoc questionnaire
TSN program use and understanding	TSN users	Ad hoc survey	Ad hoc questionnaire
TSN implementation and development indicators	TSN users	IS^a^ Extraction	TSN programs ID^b^
TSN implementation, barriers and facilitating factors	TSN implementers & stakeholders	Qualitative analyse	Semi-structured interviews
Axis 2 – TSN projects effectiveness on pathway quality			
Prevalence of hospital admissions appropriateness	HS^d^ users	Ad hoc survey	AEPf questionnaire
Number of per day hospital emergency entries	HS users	IS Extraction	SNIIRAM^c^
Prevalence of drug over- or misuse in the elderly	HS users	IS Extraction	SNIIRAM
Potentially avoidable hospitalizations	HS users	IS Extraction	SNIIRAM
Prevalence of chronic diseases	HS users	IS Extraction	SNIIRAM
Incidence of chronic diseases	HS users	IS Extraction	SNIIRAM
Incidence of disorders reflecting management pathway breaks	HS users	IS Extraction	SNIIRAM
30-day hospital readmission rate after a first hospital admission	HS users	IS Extraction	SNIIRAM
TSN use satisfaction and TSN pathway experience	TSN users	Ad hoc survey	Ad hoc questionnaire
Perception of TSN pathway contribution to patient pathway	TSN users	Qualitative analyse	Semi-structured interviews
Axe 3 – TSN projects effectiveness on professional coordination practices			
GPs’ activity volume	HS users	IS Extraction	SNIIRAM
Percentage of home visits in GPs’ activity	HS users	IS Extraction	SNIIRAM
Percentage of patients with GP noted as referent in GPs’ patient list	HS users	IS Extraction	SNIIRAM
GPs’ productivity (activity/estimated hours available)	HS users	IS Extraction	SNIIRAM
Percentage of patients with chronic conditions in GPs’ patient list	HS users	IS Extraction	SNIIRAM
Mean duration between two consecutive consultations of a same patient	HS users	IS Extraction	SNIIRAM
Percentage of night and bank holiday home visits in GPs’ activity	HS users	IS Extraction	SNIIRAM
Number of prescriptions of nursing care or physiotherapy in GPs’ activity	HS users	IS Extraction	SNIIRAM
Health professionals’ coordination practices	Health care prof users	Qualitative analyse	Semi-structured interviews
Health professionals’ participation level to TSN program	Health care prof users	Qualitative analyse	Semi-structured interviews
Axe 4 – TSN programs efficiency			
Health care expenditures (all types)	HS users	IS Extraction	SNIIRAM
Incidence of APA^1^ take-up Annual rate of institutionalization in APA beneficiaries originally living at home	HS users	IS Extraction	SNIIRAM
Hours of home helper visits done in people with APA living at home	HS users	IS Extraction	SNIIRAM
Average level of co-payment amongst APA beneficiaries living at home	HS users	IS Extraction	SNIIRAM
Health prevention expenditures	HS users	IS Extraction	SNIIRAM

^a^Information system, ^b^TSN program Information Database, ^c^SNIIRAM: French National Heath Insurance Database, ^d^Health system, ^1^APA is a personalized Autonomy allowance for the elderly, granted to cover home-care, nursing-care or institutional-care expenses

Extractions from the French National Heath Insurance Database (SNIIRAM) will be conducted to collect the main set of indicators for axes 2, 3 and 4. This exhaustive nationwide database is at the heart of the financing system of diagnosis, drugs and physicians in ambulatory care settings and of independent practitioners in private hospitals (essentially fee for service). It provides data on claims paid for each patient by the Social Security System and is therefore the main source of information on ambulatory setting activity and associated expenditures. This database contains patient data (age, sex, place of living, long-term and chronic diseases, date of birth, date of death, health insurance scheme, benefit of free complementary insurance for lower-income people), all consultations and visits to GPs and ambulatory care specialists (but nothing about their content), all medical technical procedures, all dispensed medical devices and drugs, all lab and diagnostic tests but not their results, and providers’ level data (their activity and sales turnover, geography, prescribing behaviour). This database is also linked with the PMSI (programme de médicalisation du système d’information/Information system medicalization program) database providing more detailed information on hospital activity and expenditure, including diagnoses and procedures. Extractions from the SNIIRAM will cover the time period from 2012 to 2017 and will focus on samples from the “TSN targeted people” population, in both TSN projects and control areas. Data will also be extracted from the APA (Allocation personnalisée d’autonomie/personalized autonomy allowance) database. APA is a financial support for the elderly to cover home-care, nursing-care or institutional-care expenses.

Ad hoc surveys based on questionnaires will collect information on:

- Knowledge of TSN projects assessed at T1 (early-implementation observation) and T2 (late-implementation observation) in a sample of the “TSN targeted people” population (axis 1);
- TSN program use, ease of use, satisfaction and understanding assessed at T2 in a sample of the “TSN professional users” population (axis 3);
- TSN use satisfaction and TSN pathway experience assessed at T2 in a sample of the “TSN patient users” population (axis 2);
- Appropriateness of hospital admissions assessed using the validated French version of the Appropriateness Evaluation Protocol questionnaire (AEP) in most emergency services of the TSN and control areas, twice, before TSN program implementation (T0) and at early TSN program implementation (T1) (axis 2). The survey will be carried out by one clinical research assistant assessing the appropriateness of hospital admissions of the same week day for each TSN project area and its control area.

Qualitative analyses using semi-directive interviews and focus groups will be conducted in the five TSN project areas by a team of sociologists. Three samples will be included: a sample of around 50 TSN professional users interviewed at T0, T1 and T2, for the exploration of professionals’ coordination practices, their evolution and the level of TSN projects use and adoption by professionals (axis 3); based on a biographical approach (*Illness narrative*), a sample of 10 TSN projects patients-users interviewed monthly after TSN project implementation for the exploration of users’ participation to TSN projects and the analysis of the TSN program’s contribution to patient pathway (axis 2); and a sample of the TSN national program and the five TSN projects developers and main stakeholders interviewed at T1 to analyse the way projects have been implemented, what worked well and their barriers and difficulties. Patients, TSN professionals and TSN project developers will receive incentives for their participation to qualitative analyses. The sample size of 3 × 50 participants have been decided to favor heterogeneity of health-care professionals recruited. At each step (T1, T2 and T3), 50 health-care professionals will be recruited from the five TSN territories and from the five health- and social-care professional categories: general practitioner, private-sector specialist physician, private-sector nurse, physicians working at hospital, social care professional. Sample should also be heterogeneous according to TSN program implication.

A set of standardised TSN implementation indicators will be requested to TSN projects producers and extracted from each TSN project’s database.

### Control territories

Each control territory is chosen to be as similar as possible to each corresponding TSN project territory, based on simple quantitative indicators. The identification method has already been used in France for the evaluation of another public policy (implementation of a minimal income for low-income people). Each control territory is chosen in the same region as the TSN project territory to control for regional policy effects. It must have roughly the same size as the TSN project territory, be a compact geographical zone and not be too geographically close to the TSN project territory, in order to avoid contamination. The selection process, described elsewhere, is carried in three steps:

1. Constitution of a list of candidate control territories, according to geographic, demographic and healthcare offer criteria;
2. Sorting of the list according to the territory’s structural similarity with the TSN project territory, based on a set of 20 criteria classified in four categories: 1) population demographic characteristics influencing healthcare demand; 2) population social and economic characteristics; 3) population health and 4) healthcare offer. The similarity analysis is based on factorial analyses;
3. Determining the territory that is the most similar to the TSN amongst the subset of candidate territories, according to the number of emergency room visits’ evolution, which is potentially influenced by TSN projects effects. This analysis is based on time series models. Were also considered in the final choice: feasibility of data collection and existence of competitive interventions in the territory.

### Data analysis and interpretation

Analysis involves quantitative, qualitative and data visualisation approaches.

Quantitative analyses in axes 2, 3 and 4 mostly rely on a set of standardized indicators which represent the outcomes supposedly influenced by the TSN projects in quality and intensity of healthcare consumption or medical practices. These indicators are calculated on an annual or quarterly basis and compared over time (between T0, T1 and T2). Adjustment is done through direct standardization depending on the scope of the indicator: by age and sex for general population-based measures and, to the extent possible, by clinical and social background for indicators focused on particular populations (the elderly, patients witch chronic diseases…).

Statistical estimations are then performed to assess the impact of TSN projects outcomes, with inherent limitations. First, no overall influence of TSN projects can be produced, given the variety of services provided and the disparity from one region to another: estimations are thus restricted to particular services. Second, for both technical and methodological reasons, estimation cannot be performed on subsamples of TSN users, neither for patients nor for health professionals. Instead, estimates are calculated on the whole populations targeted by the services when relevant: elderly people with functional impairments for the coordination service, young adults for vaccination reminders, patients with chronic diseases (diabetes, heart failure…) for serious games or connected devices, etc.

This approach is usually referred to as “intention-to-treat” estimate (ITT), by contrast to local average estimates (LAE) calculated on program takers/users only. Causal effects of TSN services can then be assessed if and only if i) services are exclusively intended to specific subgroups of patients, ii) these groups can be routinely identified in SNIIRAM data and iii) there is a sufficient share of actual users of the services within these groups (otherwise, variance of ITT estimate is getting too large). For axis 1 analysis, indicators of TSN use, implementation and of TSN users characteristics will be described.

Qualitative analysis of axes 1, 2 and 3 is based on interviews which are recorded, anonymous, integrally transcribed and imported into the NVivo11 software to achieve a thematic content analysis. This method aims "at spotting, in verbal or textual expressions, recurring general themes that appear under various more concrete content" [35]. This type of analysis intends to "proceed systematically in the identification, consolidation and, alternatively, the discursive examination of themes in a body"[36]. The construction process of these thematic categories, coding, is both inductive and deductive because the development of themes and sub themes rests on both literature and emerging categories of empirical analysis. The exploratory qualitative studies require in-depth exploration of these emerging categories (Grounded Theory method).

Qualitative investigations point out political and organizational barriers to implementation (axis 1), the conditions of acceptance of HI Technology and organizational innovations, the TSN program’s effects on healthcare work and professional coordination practices (axis 3), the changes in care pathway if they are significant for the patient and their family caregivers (especially patient empowerment) (axis 2).

### Monitoring and coordination

Audits of the conduct of the study are done every six months by the jury of the call, independently from the French Health Ministry. The EvaTSN project follow-up is also done yearly by an Independent Advisory Board.

### Ethical considerations

The complexity of the evaluation implies separate approval for different facets. This study has been approved by the French National Institute of Health Data (IDS – Institut des données de santé). Approval by the national CCTIRS (Comité consultatif sur le traitement de l'information en matière de recherche dans le domaine de la santé) and the CNIL (Commission nationale Informatique et Libertés) has been obtained for SNIIRAM and for qualitative analyses.

Participants in qualitative analyses (semi-directive interviews) are guaranteed strict confidentiality of records and of all statements through an encrypted identifier for each participant. Each participant fills in an informed consent form before the beginning of the study, given to the sociologist in charge of the qualitative analyses. The files and audiorecordings are kept in a sealed location of the research centre, with access limited to the qualitative analysis researchers of the EvaTSN research group, and will be destroyed after 15 years. No name will appear on any public documents and no information will be divulged that could allow participants to be identified by a third party. The EvaTSN research group members have access to anonymous quantitative datasets. All the quantitative analyses and quantitative data collection from questionnaire or databases are strictly anonymous.

Results will be disseminated as a report to the French Health Ministry. An disseminating plan to participants is in discussion with the French Health Ministry. Authorship of articles is defined according to the Vancouver Convention.

## Discussion

We expect the project to add information on HI technology effectiveness, efficiency, and effectiveness determinants in the French healthcare system. It should produce key elements for HI technology effective development and for structuring further evaluation of HI technology impact.

Many authors point out that there is a lack of evidence-based HI technology which is much needed [21, 24, 32]. Results from these studies could pave the way to a strong evidence-based implementation strategy of digital technologies in a local system, aiming at providing patient-centred and quality-assured coordinated care.

Moreover, many authors acknowledge the lack of care coordination in developed countries and the need to build and test interventions to improve care coordination [37]. Some trials testing the impact of HI technology intervention on care coordination are ongoing or already carried out in American and European countries, focusing on several conditions (depression, cardiovascular disease, cognitive impairment) [38–40]. But many HI technology interventions still need to be designed and tested in a variety of settings. Care coordination is the next opportunity and challenge for HI technology. Recently, a call to methodologically robust research on HI technology impact on care coordination was launched by the International Medical Informatics Association (IMIA) [24]. Our research project is the first one conducted in France which covers such a large perimeter of types of care and disorders or patient conditions.

Since complex interventions like HI technology include multiple components that are interrelated or interdependent, it can be challenging to develop, document, evaluate and report on them [41]. Our project will apply the principles of complex intervention evaluation, combining quantitative and qualitative analyses, conducting impact and process evaluation with multiple evaluation criteria collected from different sources (“TSN targeted people”, “TSN patient users”, “TSN professional users”, TSN developers and stakeholders, French National Heath Insurance Database). Process evaluation is essential for different reasons. It allows the understanding of the right contexts for HI technologies and their components to serve as an essential support to the delivery of patient-centred, coordinated, and quality-assured care. It also allows the evaluation of organizational changes that are paramount for the impact of new HI technology tools.

Impact evaluation using different dimensions is also needed. We evaluate the effect of TSN projects on final and intermediate health outcomes (for example: prevalence of drug overuse or misuse, prevalence of hospital admissions’ appropriateness, incidence of chronic diseases, etc.) and on patient satisfaction and experience with HI supports and coordination [42].

TSN projects are developed in five territories with rather similar size (about 200 000 inhabitants) and social profiles (high proportion of low-income population). They are geographically spread in five regions on the whole French territory and have both rural and urban areas. They represent the geographical diversity of the French territory. This may favour a good external validity of the study.

This complex evaluation has many methodological challenges.

We have to develop a common evaluation framework for five TSN projects with several HI technology components targeting different populations. This implies to have a good knowledge of all the TSN projects components, to identify the common core components of TSN projects, to have a good understanding of their action mechanisms and to rely on many dimensions (structure, process, and outcome) covering the whole spectrum of potential TSN projects’ effects. This diversity may improve mainly the external validity of our conclusions thus allowing the identification of the most effective HI technology interventions.

Another methodological challenge is linked to the gap between the evaluation and the TSN project intervention level. Each TSN project is focused on specific conditions or diseases (specific chronic conditions, social vulnerability situations, elderly dependant people, etc.) and includes samples of patients and people presenting these conditions (“TSN patient users”). Our main quantitative analyses use data from the French National Heath Insurance Database (SNIIRAM database). As it is impossible to identify the TSN patient users individually speaking, we need to select groups of people presenting similar characteristics as those targeted in the TSN projects and living in the TSN territory (“TSN targeted people”) using validated algorithms. This lack of specificity in TSN projects evaluation may lower the strength of the association between TSN projects interventions and outcomes. However the estimates correspond to the real impact assessment of TSN project at a moderate development stage. Only qualitative analyses can precisely and specifically identify and interview TSN projects users (“TSN patient users” or “TSN professional users”), in complementary means of quantitative analyses.

The third methodological challenge is linked to the controlled before-after study design. We make the strong hypothesis of the comparability of each TSN project territory with its control at the beginning and throughout all the TSN projects implementation and evaluation. We use a precise and proven method to identify the control TSN project territories, based on structural and social similarity. However, a strong hypothesis is that the control and the TSN project territories are subject to the same external factors that may influence evaluation criteria.

We face other difficulties. First, the French Health Ministry has given a short schedule. First conclusions should be given in 2017 and the evaluation should be finished in 2018. It is a very short time as the TSN projects are still being implemented and moreover, parts of some projects are in ongoing development. It is a real difficulty for the production of conclusions about TSN program health impact because we will not be able to see all the TSN projects' effects yet at that time. The HI technology evaluation model will be however described. Second, the TSN projects are still being developed although the evaluation has already started. This situation allows time for the before evaluation and leads to more contacts with TSN projects developers; it is an advantage for the precise understanding of the TSN projects components and action mechanisms. However, it is delaying the outcome and process evaluation criteria definition. Finally, the consortium has to be very careful, when dealing with interlocutors at the Ministry to keep full independence regarding strategic and methodological issues.

EvaTSN is a challenging French national research project for the production of evidenced-based information on HI technologies impact and on the context and conditions of their effectiveness and efficiency. We will be able to support health care management and policy decision making in order to implement HI technologies.

We will also be able to produce an evaluation toolkit for HI technology evaluation. This toolkit will consist in adequate study design and schedule for evaluation, appropriate evaluation criteria, data collection sources and means. It will be based on our EvaTSN large HI technology program evaluation experience.

## Abbreviations

AEP: Appropriateness evaluation protocol
APA: Allocation personnalisée d’autonomie
CCTIRS: Comité Consultatif sur le Traitement de l’Information en matière de Recherche dans le domaine de la Santé
CNIL: Commission Nationale Informatique et Libertés
EvaTSN: Evaluation Territoire de Soins Numérique
GPs: General practitioners
HI technology: Health information technology
IDS: Institut des Données de Santé
IMIA: International Medical Informatics Association
ITT: Intention to treat
LAE: Local average estimate
PMSI: Programme de Médicalisation du Système d’Information
SNIIRAM: French National Health Insurance Database
TSN: Territoires de Soins Numérique
WHO: World Health Organization
